# Notes and Comments

**Published:** 1897-05

**Authors:** 


### Notes and Comments.[1]

Relation of Tuberculous Glands in the Neck to Dental Caries.—In a report made in the *British Journal of Dental Science*, Dr. Strack gives some observations made upon over one hundred infected children, and claims to have established a distinct relation-

---

[1] The assistant editor solicits contributions for this department,—new methods, new remedies and formulas, or any short practical note which may prove of value to the practitioner or student. Address 1718 Walnut Street, Philadelphia.

ship between lymphadenoma and dental caries in forty-one per cent. of cases. In two cases he succeeded in discovering the presence of the tubercle bacillus in the tissues situated between the roots of a molar in direct contact with diseased glands. He considers it most important in treating these cases to extract all carious teeth, and in every way to place the oral cavity in a perfectly healthy condition.

---

Aluminum Crowns.—The following statement is made in the *British Journal of Dental Science.* An aluminum cap was fitted to a root, the cusps re-enforced with amalgam, and the crown immediately cemented on with oxyphosphate of zinc. The patient almost immediately complained of a sour, metallic taste. The same day he returned, the crown feeling and tasting very unpleasant. Upon entering the office he pressed the crown with his tongue, when it came off, and in lifting it from his mouth it immediately became so hot that he could only hold it by letting it drop from one hand to the other rapidly. After a short time the heat subsided, when the crown was found to be riddled with holes.

---

The Benefit of Sunlight.—Dr. T. B. Welch in his unique journal says of sunlight that it is as good a medicine for the invalid as it is a luxury to the healthy. A sun bath is a wonderful tonic, even to one who is too sick to walk out in it. The sick should, if possible, occupy the sunny side of the house, with plenty of sunlight coming immediately on the bed. Seek the sunlight is the advice of all present-day hygienists. Patients on the sunny side of the hospital ward recover soonest. The person who takes the sunny side of the street outlives his shade-seeking brother by many years. Sleep in rooms in which the sun has shed its rays during the day. And by all means the dentist should have his office so situated as to receive the sunlight during a portion of the day at least. In fact, better to have the light too strong in our offices than not enough, as the former can be modified with colored shades. If we receive sufficient sunlight we seldom need much medicine.

---

Balsamo del Platto.—In writing upon this subject in the *Dental Digest*, Dr. Davis, of Denver, Col., says that this material is just as efficient as balsamo del deserto; in fact, is quite the same thing excepting that it is found in another locality,—along the shores of the Platte River.

To prepare balsamo del platto, take carbolic acid one part, oil of cassia two parts, oil of winter-green three parts. Then take the solid balsam, melt by heat, add some vaseline, and then add enough of the above formula to keep the balsam in a thick liquid condition, about the consistency of honey, when cold. It will have an aromatic odor, yellowish color, and will be very adhesive, even sticking to moist surfaces. In root-filling follow with a gutta-percha cone. For lining cavities, dissolve the balsam in chloroform or alcohol and use as a varnish.

